# Towards a multi-physics modelling framework for thrombolysis under the influence of blood flow

**DOI:** 10.1098/rsif.2015.0949

**Published:** 2015-12-06

**Authors:** Andris Piebalgs, X. Yun Xu

**Affiliations:** Department of Chemical Engineering, Imperial College London, South Kensington Campus, London, UK

**Keywords:** thrombolysis, mathematical model, multiscale, fibrin clot, tPA, blood flow

## Abstract

Thrombolytic therapy is an effective means of treating thromboembolic diseases but can also give rise to life-threatening side effects. The infusion of a high drug concentration can provoke internal bleeding while an insufficient dose can lead to artery reocclusion. It is hoped that mathematical modelling of the process of clot lysis can lead to a better understanding and improvement of thrombolytic therapy. To this end, a multi-physics continuum model has been developed to simulate the dissolution of clot over time upon the addition of tissue plasminogen activator (tPA). The transport of tPA and other lytic proteins is modelled by a set of reaction–diffusion–convection equations, while blood flow is described by volume-averaged continuity and momentum equations. The clot is modelled as a fibrous porous medium with its properties being determined as a function of the fibrin fibre radius and voidage of the clot. A unique feature of the model is that it is capable of simulating the entire lytic process from the initial phase of lysis of an occlusive thrombus (diffusion-limited transport), the process of recanalization, to post-canalization thrombolysis under the influence of convective blood flow. The model has been used to examine the dissolution of a fully occluding clot in a simplified artery at different pressure drops. Our predicted lytic front velocities during the initial stage of lysis agree well with experimental and computational results reported by others. Following canalization, clot lysis patterns are strongly influenced by local flow patterns, which are symmetric at low pressure drops, but asymmetric at higher pressure drops, which give rise to larger recirculation regions and extended areas of intense drug accumulation.

## 1. Introduction

According to the World Health Organization [1], ischaemic heart disease and stroke accounted for over 20% of global mortality rates in 2012 and remain particularly prevalent in high-income countries. These cardiovascular disorders are often caused by thromboembolisms whereby thrombotic clots block blood supply to important tissues. These need to be treated within a very short time window in order to prevent ischaemia and excessive tissue necrosis. Occluding blood clots can be lysed through intravenous infusion of a general class of drugs called plasminogen activators in a treatment known as thrombolytic therapy [2]. However, since the drug cannot discriminate between a healthy haemostatic clot and a thrombus, the treatment can provoke internal bleeding. It has been shown that stroke patients who are treated using this method within 3 h of symptom onset have a 5% chance of developing intracerebral haemorrhage [3,4].

Clot lysis is a very complex process that is influenced by blood flow, protein reaction kinetics, clot structure and the presence of leucocytes and endothelial cells [5]. It occurs over a wide span of length and time scales thus making it a very challenging problem to model. A complete model of clot lysis would have to take into account the effect of blood flow (macroscopic effects), the movement and interaction of platelets (mesoscopic effects) and the dissolution of fibrin fibres (microscopic effects). Existing mathematical models of clot lysis can be classified into two types: continuum models with a focus on macroscopic effects and stochastic models describing micro-scale phenomena.

Diamond & Anand [6] were one of the first to model clot lysis by solving a set of reaction–diffusion–convection equations which describe the spatial movement of thrombolytic agents and other important proteins over time. In their work, the occluding blood clot was treated as a fibrous porous medium (a porous medium composed of fibrin strands) with its permeability and porosity being determined by the fibre radius. Clot lysis was modelled as a homogeneous shrinking of the fibre radius over time and Darcy's Law was used to describe fluid flow (assumed to be one dimensional) inside the clot. This model was subsequently improved to account for the effect of clot lysis inhibitors, such as *α*_2_-antiplasmin (AP) and plasminogen activator inhibitor (PAI), and to simulate the dissolution of a rectangular clot [7,8]. The latter involved changing the key properties of the clot (voidage and permeability) in each computational cell to a random value within a certain physiological range. From this they were able to observe the occurrence of lytic fingering at clot sites with a lower fibrin density. One of the limitations of their model was that it only considered transport of proteins inside the clot and neglected the effect of the surrounding blood flow. Zidansek *et al.* [9] obtained similar finger-like lysis patterns by simulating the lysis of a fully occluded clot under diffusion limited transport using a Hele–Shaw random walk model.

Wootton *et al.* incorporated the effect of outer convection [10] by solving the continuity and Navier–Stokes equations for blood flow. Resistance to blood flow within the thrombus was simulated by adding a Darcian source term to the fluid momentum equations. In their study, the transport equations were reduced to one-dimensional reaction–diffusion equations using dimensionless scaling to limit the computational cost, while the reaction kinetics were modified to take into account the presence of fibrin degradation products (FDPs) which arise from clot dissolution. This model was applied to the lysis of mural clots (fragments of clot stuck to the arterial wall) in a semi-occluded channel and their results showed that clot dissolution was accelerated by convective blood flow. Pleydell *et al.* [11] developed a steady-state analytical model to investigate spatial concentration profiles of lysis proteins along the axial and radial directions of a clot remnant found in post-thrombolysis. They observed faster clot dissolution at the leading edge of the clot and a gradually reduced dissolution rate along the length of the clot.

Recently, stochastic models have been suggested as a method to analyse the effects of clot structure on thrombolysis [12]. Bannish *et al.* created a three-dimensional multi-scale stochastic model in order to investigate how the lysis of coarse clots differs from that of fine clots [13]. Detailed biochemical reaction kinetics for the lysis of a single fibrin fibre were specified on the micro-scale, and this information was then extrapolated to determine the overall lysis of the clot on the macro-scale. They found that the difference in lytic time for the lysis of a coarse and fine clot can be accounted for by the amount of fibrin fibres in the clot and the amount of tPA molecules present per surface area of fibre. Bajd & Sersa [14] constructed a stochastic coarse-grained model of micro-scale blood clot fragmentation in order to investigate the effect of blood flow on the rate of clot lysis. Applying Newton's laws of motion to spherical objects attached by up to 20 elastic bonds, the authors showed that higher plasma flow gave rise to increased clot fragmentation, resulting in clot fragments of different sizes. This is consistent with the results of Sersa *et al.* [15] that turbulent flow accelerates clot lysis when compared to laminar flow.

The overall aim of the current study is to develop a multi-physics model for the dissolution of fibrin clots in scenarios representative of thrombolytic therapy. This requires a robust computational model that is capable of simulating clot lysis from the initial diffusion-limited transport stage to reestablishment of blood flow and subsequent mural clot lysis post-canalization. In this work, the original kinetics and fibrin microstructure model of Diamond & Anand [6] have been modified to include the solubilization of bound species during clot lysis. The effect of permeation on clot lysis is incorporated through continuity and momentum equations for the blood with a Darcian term to describe the viscous resistance of the clot to blood flow. This is implemented in a different way from the work of Wootton *et al.* [10] in that our model couples the full convection–diffusion transport equations with equations describing the reaction kinetics and blood flow and that the effect of lysis on blood flow is accounted for by including a sink term in the mass balance equation. Furthermore, unlike previous studies, we examine the entire lysis process, from the dissolution of a fully occluding clot to the lysis of the mural clots that remain after canalization.

The capability and robustness of our clot lysis model are demonstrated through application to a simplified model of an artery with an occlusive clot under varying pressure drops. By incorporating the effect of convective blood flow, the evolution of lysing pattern, the shape and subsequent lysis of remnant clots that typically arise in thrombolytic therapy can be better understood. Furthermore, the model is not limited to two dimensions; it can be applied to realistic geometries reconstructed from medical images. The model can also be used to determine the likelihood of the occurrence of secondary occlusions from the lysis of a single occluding blood clot. This in turn could help develop more effective methods in preventing arterial reocclusion following treatment for ischaemic stroke or myocardial infarction.

## 2. Material and methods

In the following section, we present the kinetic equations that govern a well-mixed model of fibrinolysis similar to the one described by Diamond & Anand [6]. After this, we provide an extension to the model to account for convection and spatial heterogeneity.

### 2.1. Adsorption and reaction kinetics of fibrinolysis

**Figure 1.**
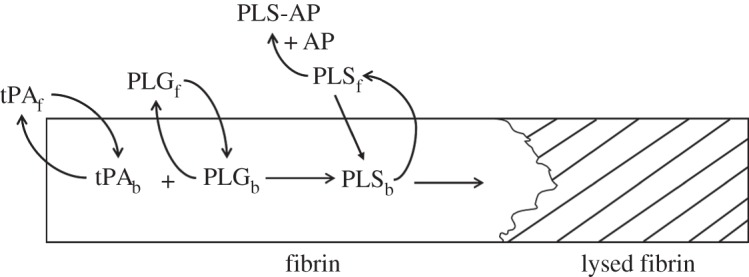
Kinetic pathway of fibrinolysis.

The kinetic pathway for fibrinolysis can be described by the interplay of three different proteins: plasmin (PLS), plasminogen (PLG) and a plasminogen activator (PA). Upon introduction of tissue plasminogen activator (tPA), which is commonly used in fibrinolysis [16], it reacts with the protein plasminogen to form the enzyme plasmin. The plasmin protease then goes on to dissolve the blood clot by cleaving its fibrin fibres. Because tPA is fibrin-specific, it needs to be adsorbed onto the fibrin surface with plasminogen in order to generate plasmin as illustrated in figure 1.

The rate of adsorption of a species onto a fibrin surface can be described by the following equation:
2.1
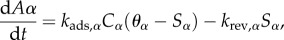
where *S_*α*_* and *C_*α*_* are the adsorbed and free phase concentrations of a species *α*, respectively, *θ*_*α*_ is the concentration of total binding sites available for adsorption on the fibrin surface, *A_*α*_* refers to the rate of adsorption of a species *α* and *k*_ads,*α*_ and *k*_rev,*α*_ are the reaction rate coefficients for adsorption and desorption, respectively. The species *α* can refer to either PLS, PLG or tPA.

For a well-mixed system, the rate of change of the free phase and bound phase concentrations of a species can be modelled as
2.2
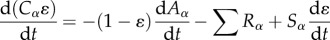
and
2.3
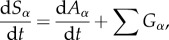
where *ɛ* is the voidage, *R_*α*_* is the rate of consumption of species *α* in the free phase and *G_*α*_* is the rate of generation of species *α* in the bound phase. The voidage represents the volume fraction of the clot occupied by the fluid. Equation (2.2) can be used to obtain the free phase concentration by solving for the superficial concentration *C*_*α*_*ɛ*. The two main bound phase reactions occurring in this study are the proteolytic cleavage of the plasminogen protein to form the plasmin protease and the fibrinolytic degradation of fibrin fibres.

Plasmin in the adsorbed phase is produced by the reaction between bound plasminogen and bound tPA. Plasmin generation follows Michaelis–Menten kinetics [17] and can be expressed as:
2.4
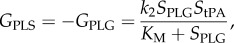
where *k*_2_ and *K*_M_ are the Michaelis–Menten coefficients required to determine the rate of plasminogen consumption and plasmin production.

A variety of clot lysis inhibitors are present in the bloodstream that can reduce the rate of fibrinolysis. The main ones are PAI, *α*_2_-AP and *α*_2_-macroglobulin (MG) [18]. However, as MG is only very weakly effective at removing plasmin from the free phase [18] and PAI only occurs in the blood circulation at low concentrations [19], their effect on clot lysis has been neglected.

The free phase plasmin is inhibited by AP and can be expressed as:
2.5
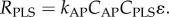


### 2.2. Quantifying clot lysis

Although it is recognized that fibrin fibres degrade through transverse cutting as observed by Collet *et al*. [20] rather than a homogeneous shrinking of the fibre radius, the latter is assumed in this study and is based on Diamond & Anand's microstructure model for fibrin fibre degradation. In order to check the validity of this assumption, results for a one-dimensional diffusion limited transport case are compared with those from Bannish *et al.* [12], who constructed a one-dimensional continuous model that takes into account the lateral transection of fibrin fibres by plasmin.

The full equations of the fibrin microstructure model have been described in detail by Diamond & Anand [6] and are presented again in the electronic supplementary material for completeness. Lysis is assumed to proceed via a homogeneous shrinking of the fibrin fibre radius, which then goes on to change the properties of the porous medium such as its voidage, the concentration of binding sites and the permeability of the clot.

The extent of clot lysis can be described by the following:
2.6
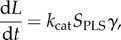
where *L* is the amount of fibrin lysed, *k*_cat_ is the reaction rate constant associated with clot lysis and *γ* is the solubilization rate (number of cuts required by PLS to cleave one unit of fibrin). The concentration of fibrin lysed can then be used to express the change in fibrin fibre radius *R_f_*(*L*), voidage *ɛ*(*R_f_*), binding site density *θ*_*α*_(*R_f_*) and clot permeability *k*(*ɛ*). These variables are then used in the macroscale transport as well as the mass and momentum balance equations. As the clot is gradually lysed, the bound terms on the fibrin fibre will be solubilized and return to the free phase. The solubilization term takes the form
2.7
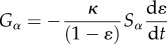

2.8
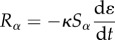
where *κ* is the solubilization constant. These two terms ensure that there are no bound phase concentrations present when the clot has been completely lysed.

### 2.3. Including the effects of convection and transport outside the clot

In order to describe the movement of blood through a porous medium, a mass and momentum balance over an arbitrary control volume needs to be taken. As the configuration of the porous–fluid interface can be very complex, a volume averaging method to derive the equations of fluid flow was used [21,22]. Taking a mass and momentum balance over this control volume gives the following equations for fluid flow written in Einstein summation notation:
2.9
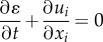

2.10
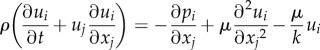
where *ρ* is the density of blood, *u* is the velocity, *p* is the pressure, *µ* is the viscosity and *k* is the permeability. The fluid is assumed to be incompressible, Newtonian and the clot is assumed to be homogeneous and isentropic.

The well-mixed kinetics described in §2.1 can then be extended to include spatial variations in concentration. The following expression, written in Einstein summation notation (see the electronic supplementary material for more information), evaluates the temporal and spatial changes in concentration for any species in the free phase:
2.11
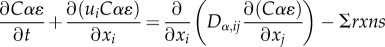
where *D_*α*,ij_* refers to the hydrodynamic dispersion and *Σrxns* represents the well-mixed kinetic terms defined on the right-hand side of equation (2.2). In this study, we have assumed that diffusion is isotropic and constant throughout the domain. Furthermore, as the free phase species are now spatially varying, the bound phase species (represented by equation (2.3)) become a function of *x*, *y* and *t*.

### 2.4. Numerical procedures and model details

First, the well-mixed kinetics described by equations (2.1)–(2.8) were solved to evaluate the temporal change in the concentrations of tPA, PLG, PLS and AP. The system of ordinary differential equations (ODEs) was solved in Matlab^1^ using ode45 that employs a fourth-order Runge–Kutta solver. Values for all the kinetic constants used in the simulation can be found in the electronic supplementary material.

The well-mixed kinetics model was then extended to evaluate the spatial distribution of protein concentrations and clot properties for a one-dimensional diffusion-limited transport system. This was implemented by performing an explicit finite difference discretization of a simplified version of the transport equation described by equation (2.11). The second-order diffusion term was discretized using a second-order central difference scheme while the transient term was discretized using a first-order forward difference approach.

**Figure 2.**
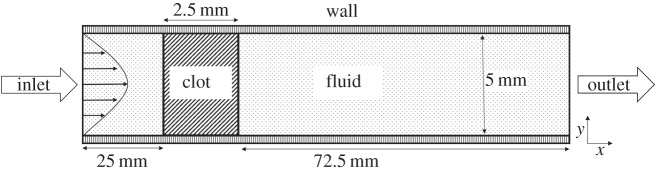
Geometry of a fully occluded artery used in the computational simulations.

Finally, the complete model including the effects of convection as described by equations (2.9) and (2.10) was implemented in ANSYS Fluent^2^ via user-defined functions (UDFs) written in the C programming language. To reduce the computational demand, only the three main components of lysis (PLG, PLS and tPA) were included. The role of AP was deemed negligible as the well-mixed kinetics model shows that in its absence, only a small amount of PLS in the free phase is present in the bloodstream and this has a negligible effect on the lysis time. The model was applied to a rectangular occlusive clot in a two-dimensional channel as illustrated in figure 2. The channel height of 5 mm corresponds to an average diameter of a cerebral artery in the brain [23], while the clot width of 2.5 mm is representative of clots typically found in ischaemic stroke [24]. The length of the channel was chosen to ensure that the model inlet and outlet were sufficiently far so as to minimize the influence of artificial boundary conditions on predicted flow patterns and concentration profiles near the clot. A structured mesh was generated and mesh sensitivity tests showed that over 200 000 elements would be required to achieve mesh independent solutions.

Regarding initial conditions, starting with the well-mixed model a physiological PLG concentration of 2 µM [7] was first introduced into the system and allowed to reach a dynamic equilibrium. The fibrin clot had an initial fibrin density of 0.28 mg ml^−1^ [25] and an initial fibrin radius of 250 nm [26]. The resulting free phase and bound phase PLG concentration was 1.65 and 34.9 µM, respectively. After this, 50 nM of tPA [7] was added to the system and monitored until the clot had been completely lysed. For the one-dimensional diffusion-limited transport model and two-dimensional convection-included model, the following boundary conditions were applied: at the inlet a tPA concentration of 50 nM and a PLG concentration of 2 µM were specified, while the outlet was located sufficiently far such that the concentration gradient is zero. In addition, the two-dimensional model had pressure values imposed at the inlet and outlet to give a constant pressure drop across the channel, with pressure drops ranging from 1 to 20 Pa based on previous experimental work [27]. Walls were specified as no-slip and non-permeable. The two-dimensional clot lysis model was initially solved for steady fluid flow with a completely occluding clot before tPA was introduced into the system. The concentration of bound PLG was set to 34.9 µM as found from the well-mixed kinetics simulation. The same kinetics parameters were adopted in all simulations unless otherwise specified.

## 3. Results

In this section, the results for the well-mixed kinetics model are given first followed by those of the one-dimensional diffusion limited transport model. After this, results obtained from the extended transport model for clot lysis in a fully occluded channel are presented for a variety of pressure drops.

### 3.1. Well-mixed kinetics model

**Figure 3.**
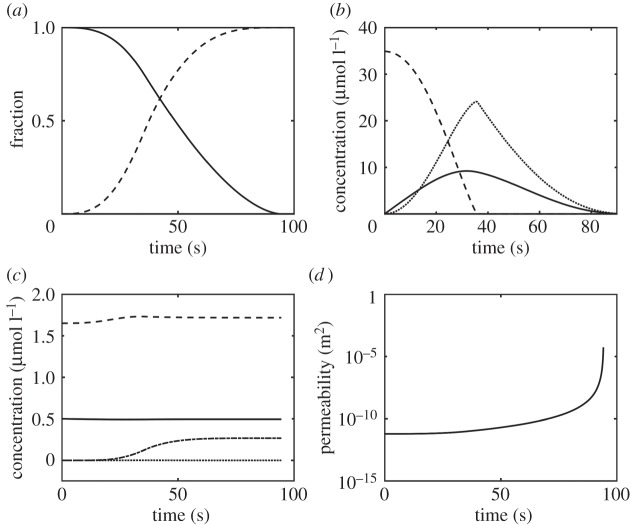
Temporal variations obtained from the well-mixed kinetics model. (*a*) Normalized fibre radius and the fraction of clot lysed. Solid line refers to the ratio R_f_/R_f0_ and the dashed line to the fraction of clot lysed; (*b*) concentrations of bound tPA ×100 (solid), PLG (dashed) and PLS (dotted); (*c*) concentrations of free tPA ×10 (solid), free PLG (dashed), free PLS (dotted) and free AP-PLS complex (dotted and dashed) and (*d*) permeability of the system that tends to infinity as the clot is completely lysed.

Figure 3 shows the concentration of each component as well as changes in the properties of the clot over time. It shows that clot lysis is completed at around 90 s upon addition of 50 nM of tPA to the system. It should be noted that the concentration of free tPA and that of free PLG are not constant, but change by a small amount over time. Close-up figures of these concentrations can be found in the electronic supplementary material.

**Figure 4.**
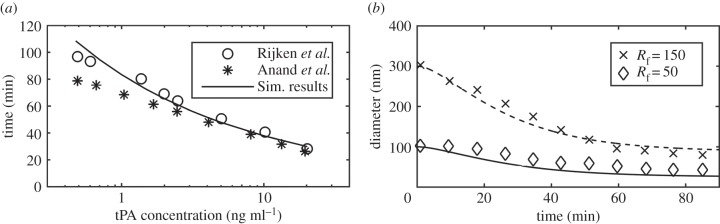
(*a*) Time taken to achieve 95% lysis in a well-mixed clot model for different tPA concentrations. Simulation results (solid line) are compared with experimental results (circles) and simulation results in the literature (asterisks) [28,29] and (*b*) change in fibrin fibre radius compared with experimental results for an initial fibrin radius of 150 nm (crosses) and 50 nm (diamonds) [26].

As a part of the validation exercise, simulations were repeated to replicate the experimental and computational conditions found in the literature [28,29]. Figure 4*a* shows the comparison of minimum time required to achieve 95% lysis in a well-mixed system for a clot of a fibrin concentration of 5.88 µM for different tPA concentrations. The values of *K*_M_ and *k*_2_ were changed to 2.42 µM and 0.22 s^−1^ to match the experimental conditions [28]. Figure 4*b* shows the comparison of change in fibrin radius between our prediction and experimental data [26], for which an extra consumption term was introduced to account for the loss of plasmin activity over time. Further details of the experimental conditions and parameters used can be found in the electronic supplementary material.

### 3.2. One-dimensional diffusion model

**Figure 5.**
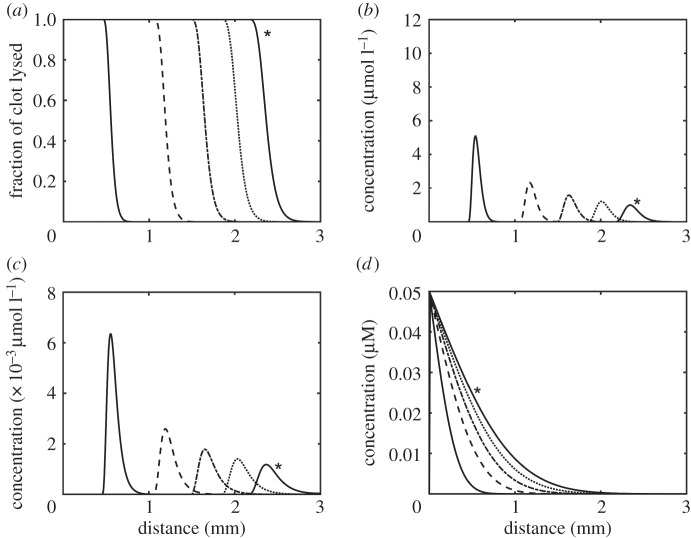
(*a*) Movement of the lysis front, (*b*) concentration of bound PLS, (*c*) concentration of bound tPA and (*d*) concentration of free tPA. The solid, dashed, dashed with dot, dotted and solid with asterisk lines for each plot correspond to the value of the variables in each plot at 10, 30, 50, 70 and 90 min, respectively.

**Figure 6.**
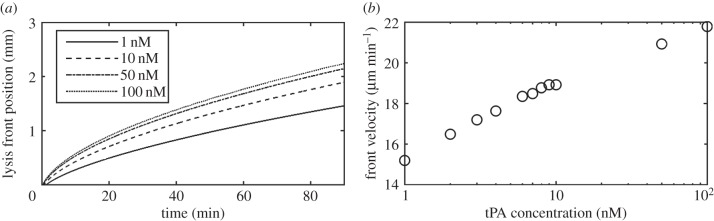
(*a*) Lysis front position over time for different tPA inlet concentrations. The lysis front position was calculated as the farthest *x* node that has reached 95% lysis and (*b*) change in the lysis front velocity for different tPA concentrations. The average velocity was taken as the positive change in the lysis front position from 10 to 80 min.

Figure 5 shows spatial variations of clot properties and concentrations of the bound and free phase lysis proteins. The lysis front seems to accelerate in the initial period before travelling at a constant speed through the clot medium as shown in figure 5*a*. Figure 5*b* and *c* shows the movement of the bound tPA and PLS over time, respectively. The initial concentration profile is narrow with a sharp peak that rapidly falls off and the profile becomes wider over time. Figure 5*d* shows the free tPA gradually diffusing through the system and approaching a uniform concentration over time. Figure 6 shows the influence of tPA concentration on the lysis front position (point of 95% lysis or greater) and average front velocity. It can be seen that after an initial acceleration period, the lysis front velocity tends to remain relatively constant. Gradually increasing the initial tPA concentration at the inlet seems to give rise to modest increases in the lysis front velocity, as demonstrated by the logarithmic plot in figure 6*b*.

### 3.3. Two-dimensional simulations of clot lysis

**Figure 7.**
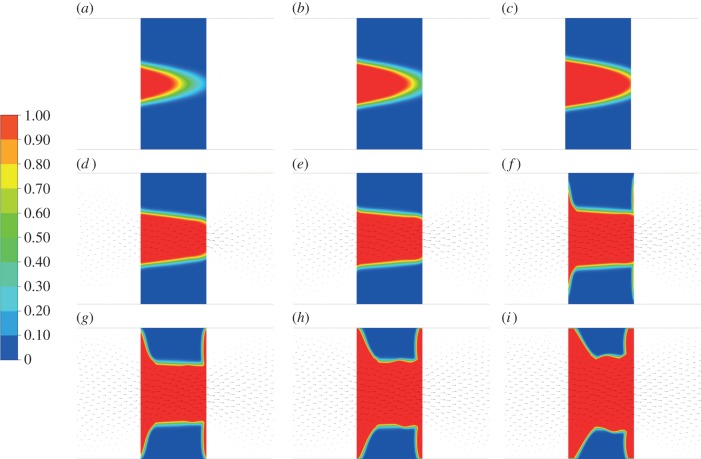
Clot lysis contours over time for a 1 Pa pressure drop. The legend on the left refers to the fraction of clot that has been lysed with red corresponding to complete lysis and blue corresponding to no lysis. (*a*) BTR = 0.50, (*b*) BTR = 0.75, (*c*) BTR = 1.00, (*d*) BTR = 1.25, (*e*) BTR = 1.50, (*f*) BTR = 1.75, (*g*) BTR = 2.00, (*h*) BTR = 2.25 and (*i*) BTR = 2.50.

Figure 7 shows the evolution of lysis contours superimposed on velocity vectors for an inlet pressure of 1 Pa and outlet pressure of 0 Pa. A dimensionless quantity, termed the breakthrough ratio (BTR), is used to investigate clot lysis similarities across different pressure drops. It is defined as the simulation time over the breakthrough time, with zero corresponding to the time when the first cell has been lysed by 1.0% and a breakthrough time being assigned when one of the cells at the clot exit reaches over 95.0% lysis. This simulation was repeated for pressure drops of 5 and 10 Pa, respectively, and the corresponding lysis contours can be found in the electronic supplementary material.

**Figure 8.**
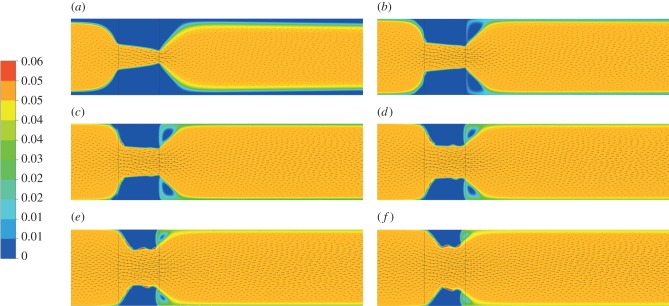
Concentration of free tPA contours for a 1 Pa pressure drop. The red regions denote areas of a concentration of 0.06 µM or higher, while the blue regions show areas with almost no tPA. (*a*) BTR = 1.25, (*b*) BTR = 1.50, (*c*) BTR = 1.75, (*d*) BTR = 2.00, (*e*) BTR = 2.25 and (*f*) BTR = 2.50.

**Figure 9.**
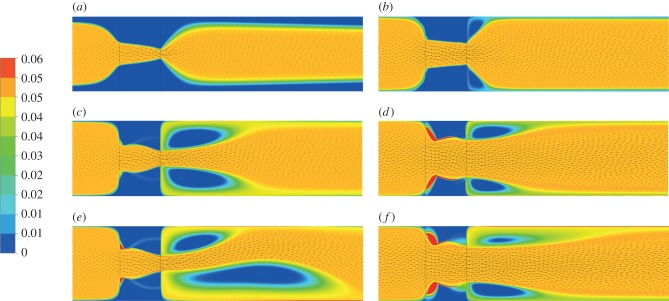
Concentration of free tPA contours for different pressure drops. (*a*) 1 Pa pressure drop, BTR = 1.20; (*b*) 1 Pa, BTR = 1.50; (*c*) 10 Pa, BTR = 1.20; (*d*) 10 Pa, BTR = 1.50; (*e*) 20 Pa, BTR = 1.20 and (*f*) 20 Pa, BTR = 1.50.

**Figure 10.**
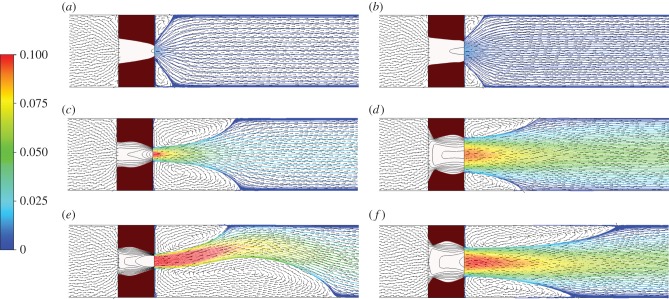
Velocity streamlines and velocity vectors for different pressure drops. The dark red region represents the amount of clot that has not been lysed. (*a*) 1 Pa pressure drop, BTR = 1.20; (*b*) 1 Pa, BTR = 1.50; (*c*) 10 Pa, BTR = 1.20; (*d*) 10 Pa, BTR = 1.50; (*e*) 20 Pa, BTR = 1.20 and (*f*) 20 Pa, BTR = 1.50.

Figure 8 shows the free tPA concentration contours, while figure 9 shows the concentration of free tPA distributed throughout the system for different pressure drops. Each row of images is for a certain pressure drop and each column of images is for a chosen BTR. To analyse the association between flow and lysis patterns, instantaneous streamlines at selected pressure drops and time points are given in figure 10.

## 4. Discussion

In this paper, we present a multi-physics model that is capable of simulating blood clot lysis in a macroscale artery upon introduction of tPA. The occluding blood clot was modelled as a porous medium with its properties varying as a function of the bound PLS concentration. The transport of tPA and other proteins was modelled with a set of convection–diffusion–reaction equations while the blood flow was solved based on fluid mass and momentum balances. The coupled transport and fluid flow model was implemented in ANSYS Fluent and can be applied to realistic arterial geometries.

The results of the well-mixed model show that lysis reaches completion at around 90 s, which is consistent with data in the literature [29] except that the lysis curve gradually tends to 100% in our model. This is due to the modified source term describing the solubilization of the bound plasmin to the free phase following clot lysis. The well-mixed simulation results have been compared with experimental data [26,28] as shown in figure 4*a*,*b*, demonstrating the validity of the kinetics model.

The one-dimensional diffusion model shows clearly that there is an accumulation of bound lytic proteins at the lysis front (figure 5*b*,*c*) which falls off as the clot is gradually lysed. This moving lysis front is consistent with experimental observations in the literature [30,31], where lysis was found to be preceded by the accumulation of lytic proteins. These results are also very similar to those reported by Bannish *et al.* [12], who constructed a continuous one-dimensional model of clot lysis taking into account the transverse cutting of fibrin fibres. The same trend of the lysis front moving gradually through the clot can be seen as well as the shape and change of bound species concentration profiles, the latter displaying a similar trend in becoming shorter and wider over time due to enhanced uniformity of free tPA spreading throughout the domain. The change in peak value is more marked in our simulation because of the larger clot width (3 mm as opposed to 0.3 mm modelled by Bannish *et al.*). The similarity in the results confirms the validity of using the Diamond & Anand fibrin microstructure model for macroscopic simulations of clot lysis.

The lysis front position and average front velocity shown in figure 6 suggest that in the low concentration range (1–10 nM) increasing tPA concentration would lead to a large increase in the lysis front velocity as reported in the literature [20]. At high concentrations, however, the gradient of the lysis front velocity appears to decrease exponentially as shown in the logarithmic plot in figure 6*b*. The values for the lysis front velocity in diffusion limited transport lie within the same range as those given in the literature. For instance, Wootton *et al.* found typical lysis front velocities at around 30 µm min^−1^ [10], Anand *et al.* at 50 µm min^−1^ and Bannish *et al.* at 20 µm min^−1^ for diffusion limited transport [7,12,13].

Having established the validity of the kinetics model and parameter values adopted in the model, we can now focus on examining the factors that influence the lysing pattern of a single occlusive blood clot. By simulating the dissolution of a fully occluding clot in a two-dimensional channel, clot lysis patterns and their evolution over time can be obtained. Figure 8 shows clearly that lysis starts at the centre of the clot and creates a funnel-shaped space that allows for lysis to proceed radially. This is consistent with the observations of Anand *et al.* [8] and Zidansek *et al.* [9] in the case of diffusion-limited transport, except for the finger-like lysing pattern which is not predicted by our model owing to the assumption of uniform initial clot properties. Upon recanalization (BTR = 1.0), lysis of the remnant clot occurs not just radially but also at the front and the back of the clot. There also appears to be a dent in the middle of the clot surface which tends to become more noticeable over time (figure 7*h*,*i*). The mechanisms responsible for these observations can be explained by examining the free tPA concentration contours given in figures 8 and 9, in conjunction with the streamline plots in figure 10.

It can be seen that following recanalization, blood flow through the centre of the clot is established. Typically, there is a high-velocity jet through the canal along the centre, but the sudden expansion of flow channel at the end of the clot causes flow to decelerate and consequently allow for the formation of flow recirculation distal to the clot. Flow recirculation helps recycle the free phase tPA to the back of the clot and allows the lysis front to develop behind the obstruction. Furthermore, there appears to be a build-up of tPA concentration at the surface in the front and in the middle of the remnant clot, especially in areas where the surface caves in thus trapping tPA in a slow-moving region.

Figure 9 shows the free phase tPA concentration contours for three different pressure drops: 1, 10 and 20 Pa. It can be seen that the value of the pressure drop has a strong influence on the distribution of free tPA throughout the domain. First of all, a higher pressure drop causes an elevation of free tPA concentration towards the front of the clot. This causes a strong lysis front pushing the dissolution of the clot forward in the direction of blood flow. Secondly, a higher pressure drop promotes the development of larger recirculation regions distal to the clot that improves mixing and accelerates the development of the lysis front distal to the clot. Lastly, at very high pressure drops, the flow becomes more disturbed as can be seen by the formation of asymmetric recirculation regions (figure 10). Naturally, these asymmetric regions cause non-uniform lysis of the remnant clot with a larger recirculation region corresponding to faster clot dissolution. How further disturbed flow may affect clot lysis patterns remains to be seen, but due to the improved mixing, it is suspected that it will accelerate clot lysis [15].

The present model has a number of limitations. Clot break-up due to breakage and erosion is not incorporated due to its high computational demand [14]. A combination of stochastic and continuous processes may help to understand the effect of clot breakage at different pressure drops. Furthermore, clot dissolution is described by fibrinolysis while in reality clots are composed of many different cells, including red blood cells and platelets. The exposure of new binding sites due to proteolytic cleavage of fibrin by plasmin and the effect of endothelial cells on clot dissolution have also been neglected. The clot dissolution kinetics only describe the dynamics of three proteins and hence do not account for clot regeneration due to thrombin activity. Lastly, fibrinolysis is described using a model based on the homogeneous shrinking of the fibrin fibre radius rather than lateral transection [20]. A continuum model of this was attempted but found to be lacking in describing the differences in the lysis time between fine and coarse blood clots [12]. A hybrid stochastic and continuous model may be required to implement this feature [13].

However, despite the assumptions involved, the model provides a multiphysics framework for the simulation of a clot lytic process in physiologically realistic geometries. The model shows that higher pressure drops across the clot can lead to secondary complications while enhancing the lysis of the clot through better mixing. Clinically, it is important to achieve arterial recanalization as soon as possible (within a few hours) of the diagnosis of a thromboembolic disease such as stroke. Treatment with thrombolytic therapy can sometimes be discarded if the patient has increased susceptibility to side effects due to certain physiological risk factors such as high blood pressure. The model results show that although lysis can proceed more rapidly at elevated pressures due to faster drug penetration, the development of disturbed flow may increase the chances of small clot fragments breaking off from the original clot. These fragments may cause secondary occlusions and can also bring about thrombosis in other areas. This may have important implications for hypertensive patients or patients who have an elevated resistance in their downstream vasculature.

In the future, we plan to improve this model by accounting for platelets in the expression of clot permeability, extending the reaction kinetics to account for more detailed biochemistry and developing a more accurate description of fibrinolysis. We would also like to use this model to examine lysis patterns that occur in physiologically realistic geometries in the hope of analysing clot lysis patterns and evaluating the effectiveness of thrombolytic therapy in treating ischaemic stroke and myocardial infarction based on patient-specific data.

## 5. Conclusion

A continuum approach multi-physics model has been developed to simulate the entire lytic process of an idealized fully occlusive blood clot. In this model, we modified the kinetics presented by Anand & Diamond by including the solubilization of bound species as clot lysis progressed, and extended the work of Wootton *et al.* [10] to allow for multi-dimensional transport. The model predictions agree well with experimental data [26,28] and computation results reported by others [10,12]. By using the modified continuity and fluid momentum equations, the effects of permeation on clot lysis could be accounted for and the effect of fluid flow on clot lysis can be evaluated. It has been found that the pressure drop across the clot has a strong influence on the patterns of clot lysis and the clot lysis time. High pressure drops allow the drug to break through the clot at a much faster rate, resulting in the formation of a thinner funnel-shaped canal at the centre of the clot. At the same time, high pressure drops also lead to the development of recirculation regions distal to the clot that promote faster clot lysis, resulting in thinner and taller remnant clots. It is possible that at higher pressure drops, the development of these recirculation regions may cause the lysis fronts at the upstream and downstream end of the clot to break off into small clot fragments. In the future, we hope to evaluate the effect of disturbed flow on clot lysis, incorporate platelets in the description of the blood clot properties and examine the lysis patterns of blood clots in more realistic geometries.

## Supplementary Material

Additional Materials

## Endnotes

1 MATLAB R2015b. The Mathworks, Natick, MA, USA.

2 ANSYS Fluent V14.5. ANSYS Inc., Canonsburg, PA, USA.
