# Supplementary material for: Towards a multi-physics modelling framework for thrombolysis under the influence of blood flow
Source: J R Soc Interface. 2015 Dec 6;12(113):20150949. doi: 10.1098/rsif.2015.0949 (PMC4707866; doi:10.1098/rsif.2015.0949)
Supplement: Additional Materials [file rsif20150949supp1.doc]

**Additional Material**

The following documentation contains extra information that the reader may find useful in reference to understanding the research article “*Towards a Multi-Physics Modelling Framework for Thrombolysis under the Influence of Blood Flow”* by Andris Piebalgs and X. Yun Xu. Here we present the nomenclature, values for model parameters, derivations of equations and additional figures not given in the research paper.

## NOMENCLATURE

The table below contains a list of definitions of symbols used in the paper.

Table A1: Nomenclature

| **Symbol** | **Meaning** |
| --- | --- |
| A | Concentration of Species Adsorbed |
| a_0_ | Amount of Fibrin Monomers per Protofibril Length |
| C | Free Phase Concentration |
| D | Dispersion Coefficient |
| G | Rate of Species Generated |
| k | Permeability |
| k_AP_ | Anti-plasmin Reaction Coefficient |
| r_0_ | Average Radius of a Protofibril |
| k_2_ | Michaelis-Menten Reaction Coefficient |
| k_ads_ | Forward Adsorption Coefficient |
| k_cat_ | Lysis Reaction Rate Coefficient |
| k_decay_ | Decay Coefficient |
| K_M_ | Michaelis-Menten Reaction Coefficient |
| k_rev_ | Reversible Adsorption Coefficient |
| L | Concentration of Fibrin Lysed |
| L_pf_ | Total Length of Protofibrils |
| MW_fbn_ | Molecular Weight of Fibrin |
| n | Permeability Coefficient |
| N_AV_ | Avogadro's Constant |
| p_0_ | Amount of Protofibril per Fibre Cross-sectional Area |
| PLG | Plasminogen |
| PLS | Plasmin |
| R | Rate of Species Produced |
| R_f0_ | Initial Radius of Fibrin Fibre |
| R_f_ | Radius of Fibrin Fibre |
| S | Concentration of Bound Species |
| S_mom_ | Momentum Source Term |
| t | time |
| tPA | Tissue Plasminogen Activator |
| u | Velocity |
| U_0_ | Volume of Computational Cell |
| γ | Amount of Cuts Required by Plasmin to Cleave One Unit of Fibrin |
| ε | Voidage |
| θ | Binding Site Concentration |
| λ | Permeability Constant |
| μ | Dynamic Viscosity |
| ρ_fibre_ | Density of fibrin in Fibre |
| ρ_β_ | Density of Fibrin in Clot |
| χ | Amount of Protofibrils Available for Lysis |

**EINSTEIN SUMMATION NOTATION**

The section below expands on the meaning of certain terms written in Einstein summation notation.

Where *η* is a vector quantity defined as

Another important summation quantity used in the study is defined as.

**TRANSPORT EQUATIONS**

In this section, the differential equations that were used for the 2D clot lysis model are presented in their full form.

**Free tPA**

**Free PLG**

**Free PLS**

**Free AP (Well-Mixed Case)**

**Free AP-PLS Complex (Well-Mixed Case)**

**Bound tPA**

**Bound PLG**

**Bound PLS**

**Concentration of Clot Lysed**

**FIBRIN MICROSTRUCTURE MODEL**

This section provides a description of the clot lysis fibrin microstructure model as outlined by Diamond and Anand (6). The blood clot is assumed to be a fibrous porous medium composed only of strands of fibrin fibres.

The fibrin mesh is assumed to be compromised of fibrin monomers that combine in a half-staggered manner to form protofibrils that go on to laterally aggregate and form fibrin fibres. The microstructure model assumes that the fibrin radius is uniform across the entire fibre and degrades homogeneously as lysis goes on. The concentration of fibrin lysed can be described by the following equation:

|  |  |  |
| --- | --- | --- |

where *L* is the amount of fibrin lysed, *k_cat_* is the reaction rate constant associated with clot lysis and *γ* is the solubilisation rate per number of cuts required by PLS to cleave one unit of fibrin. The amount of fibrin lysed can be used to evaluate the change in fibrin fibre radius

|  |  |  |
| --- | --- | --- |

where *R_f_* represents the fibre radius, *p_0_* represents the amount of protofibrils per fibre cross-sectional area, *L_pf_* is the length of each protofibril, *a_0_* is the amount of fibrin monomers per length of protofibril, *N_AV_* is Avogadro’s constant and *U_0_* is the volume of the control surface (computational cell).

For a fibrous porous medium, the radius of the fibrin fibre can be used to calculate the voidage and permeability. The voidage is expressed as

|  |  |
| --- | --- |

where *ρ_β_* is the fibrin density in the control volume and *ρ_fibre_* is density of fibrin in a fibre. The concentration of the total amount of binding sites that are sterically accessible to the proteins can be found by calculating the amount of binding sites per fibrin monomer and the total amount of these monomers in a protofibril .

|  |  |  |
| --- | --- | --- |

Where *χ* is the number of protofibrils that are sterically accessible and *q*_α_ is the number of binding sites in a fibrin monomer for species *α*. In this study, we assume that all the protofibrils in the fibrin fibre are sterically accessible.

|  |  |
| --- | --- |

where *r_0_* is the average radius of a protofibril. The derivation of this expression can be found in Additional Material. Finally, the permeability of a fibrous porous medium can be expressed as a function of the fibre radius and porosity (6,7,8).

|  |  |  |
| --- | --- | --- |

where *k* is the permeability, *n* is the permeability coefficient and *λ* is the permeability constant (chosen within the range specified by Wufsus et al.[[1]](#footnote-2)).

## TABLE OF VALUES

The following section lists the values of model parameters used in the computations presented in the research article.

### **Well Mixed Kinetic Simulation Constants**

The well mixed kinetics model defined in the paper was used to generate Figures 3 and 4. The following values for the constants were used (see Table A1 for definitions of symbols).

Table A2: Values for Model Parameters

| **Symbol** | **Value** | **Units** | **Reference** |
| --- | --- | --- | --- |
| a_0_ | 0.0444 | nm^-1^ | (6) |
| k_2_ | 15 | s^-1^ | (6) |
| k_ads,PLG_ | 1.087 x 10^-4^ | μM^-1^ s^-1^ | (29) |
| k_ads,PLS_ | 0.500 x 10^-3^ | μM^-1^ s^-1^ | (29) |
| k_ads,tPA_ | 1.148 x 10^-4^ | μM^-1^ s^-1^ | (29) |
| k_AP_ | 10 | μM^-1^ s^-1^ | (18) |
| k_cat_ | 10 | s^-1^ | (6) |
| k_decay_ | 8.45 x 10^-4^ | s^-1^ | (26) |
| K_M_ | 0.13 | μM | (6) |
| k_rev,PLG_ | 4.131 x 10^-3^ | s^-1^ | (29) |
| k_rev,PLS_ | 5.435 x 10^-5^ | s^-1^ | (29) |
| k_rev,tPA_ | 6.658 x 10^-5^ | s^-1^ | (29) |
| MW_fbn_ | 3.4 x 10^5^ | g mol^-1^ | - |
| n | 1.8 | - | - |
| N_AV_ | 6.02 x 10^23^ | mol^-1^ | - |
| p_0_ | 0.01116 | nm^-2^ | (6) |
| r_0_ | 2.39 | nm | (6) |
| R_f0_ | 250 | nm | - |
| γ | 0.1 | - | (6) |
| λ | 0.025 | - | - |
| ρ_fibre_ | 0.28 | g ml^-1^ | (6) |
| ρ_β_ | 0.003 | g ml^-1^ | - |

Note that μM is equivalent to μmoles/L.

### In order to replicate the results by Anand et al (29), we found that either the value for the binding site concentration has to be enhanced by a multiple of 50 or the kinetic constants for adsorption/desorption should be changed to those used by Wootton et al (10).

### **Finite Difference Simulation Constants**

The well mixed kinetics model was extended to analyse clot lysis in 1D diffusion limited transport. This was done to generate Figures 5 and 6 in the research article. The same kinetic parameters as those defined in Table A2 were used. Parameters related to numerical discretisation are given in Table A3.

Table A3: Values used for FD Discretisation

| **Symbol** | **Value** | **Units** | **Meaning** |
| --- | --- | --- | --- |
| D | 5 x 10^-11^ | m^2^ s^-1^ | Dispersion Coefficient |
| dt | 0.1 | s | Time-step Value |
| dx | 0.01 | mm | Spatial-step Value |
| L | 3 | mm | Clot Length |
| nt | 2.4x10^4^ | - | Number of Discrete Time Points |
| nx | 300 | - | Number of Discrete Spatial Points |
|  |  |  |  |

## Comparison with Experimental Results

We matched our numerical predictions with the experimental results given by Blinc et al (26) and Rijken et al (28). Here we outline the experimental set up of each and describe any modifications that have been made in the kinetic constants or the modelling equations.

Blinc et al (26) created different types of clots by varying NaCl concentrations and analysed their fibrin microstructure upon lysis with PLS using atomic force microscopy. The clots were created using 1.8 mg/ml fibrinogen and had a range of average fibrin diameters between 150 to 600 nm. A plasmin concentration of 0.17 U/ml was applied to 2 clots with a fibrin diameter of around 100 nm and 300 nm. Because the plasmin was in the system for a very long time (>20 mins), we added an extra degradation term to account for the denaturing of the plasmin. This is assumed to occur only in the free phase and can be represented by the following equation.

The value of *k_decay_* was obtained from Blinc et al (26) where it can be clearly seen that the majority of initial plasmin (>99%) was denatured by 80 minutes. A k_cat_ value of 1 s^-1^ was found to best fit the experimental data. All other kinetic parameters are the same as those in Table A2.

Rijken et al (28) evaluated the lytic efficacy of extrinsic tPA in its one-chain and two-chain forms. They created clots using 2 mg/ml fibrinogen in a 0.14 M NaCl solution and then evaluated the clot lysis time for varying concentrations of one-chain tissue plasminogen activator. Using the results obtained by Blinc et al (26), we assumed that the average initial diameter of fibrin fibres in the clot created in these conditions was 300 nm. The initial plasminogen concentration was 0.15 mg/mL and the kinetic parameters for plasminogen activation were K_M_ = 2.42 μM and k_2_ = 0.22 s^-1^. The kinetic value of k_cat_ = 5 s^-1^ used by Wooton et al (10) and Anand et al (6) was chosen. All other kinetic parameters are the same as those in Table A2.

## ADDITIONAL FIGURES

The section below presents the additional figures obtainable from the simulations.

**Close-up of Figure 3**

Figure A1 shows close-up images of the free-phase tPA and PLG concentrations from the well-mixed simulations shown in Figure 3.


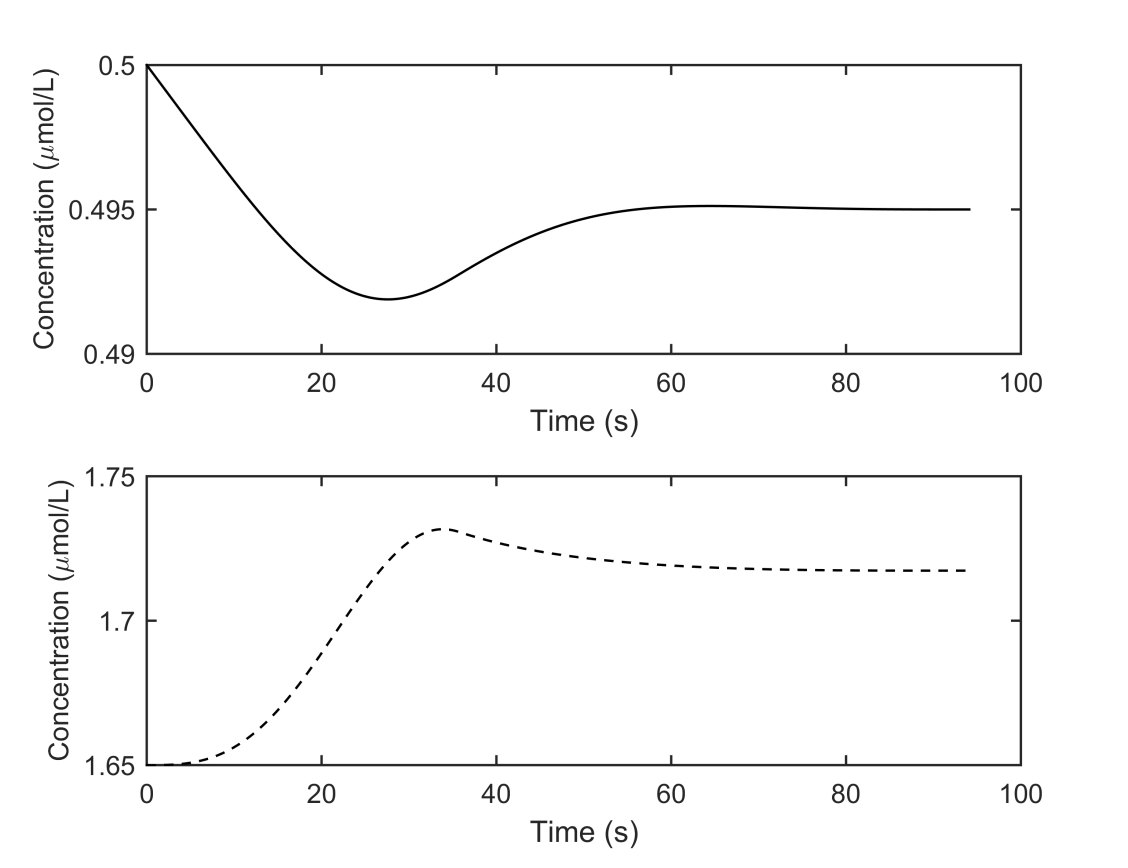


Figure A1: Free phase concentrations over time for (Top) tPA; (Bottom) PLG.

## Breakthrough Times

Table A4 shows the breakthrough times found for each pressure drop. This value corresponds to the time at which the first cell at the exit of the clot reached over 95% lysis. These values are later used for data post-processing.

Table 4: Breakthrough Times

| **Pressure Drop (Pa)** | **Breakthrough Time (s)** |
| --- | --- |
| 1 | 426.00 |
| 10 | 238.50 |
| 20 | 184.00 |

## Lysis Contours for 10 Pa and 20 Pa Pressure Drop

Figures A2 and A3 show the lysis contours obtained for a 10 Pa and 20 Pa pressure drop across the clot. The legend corresponds to the fraction of clot lysed with red corresponding to 100% lysis or complete lysis.


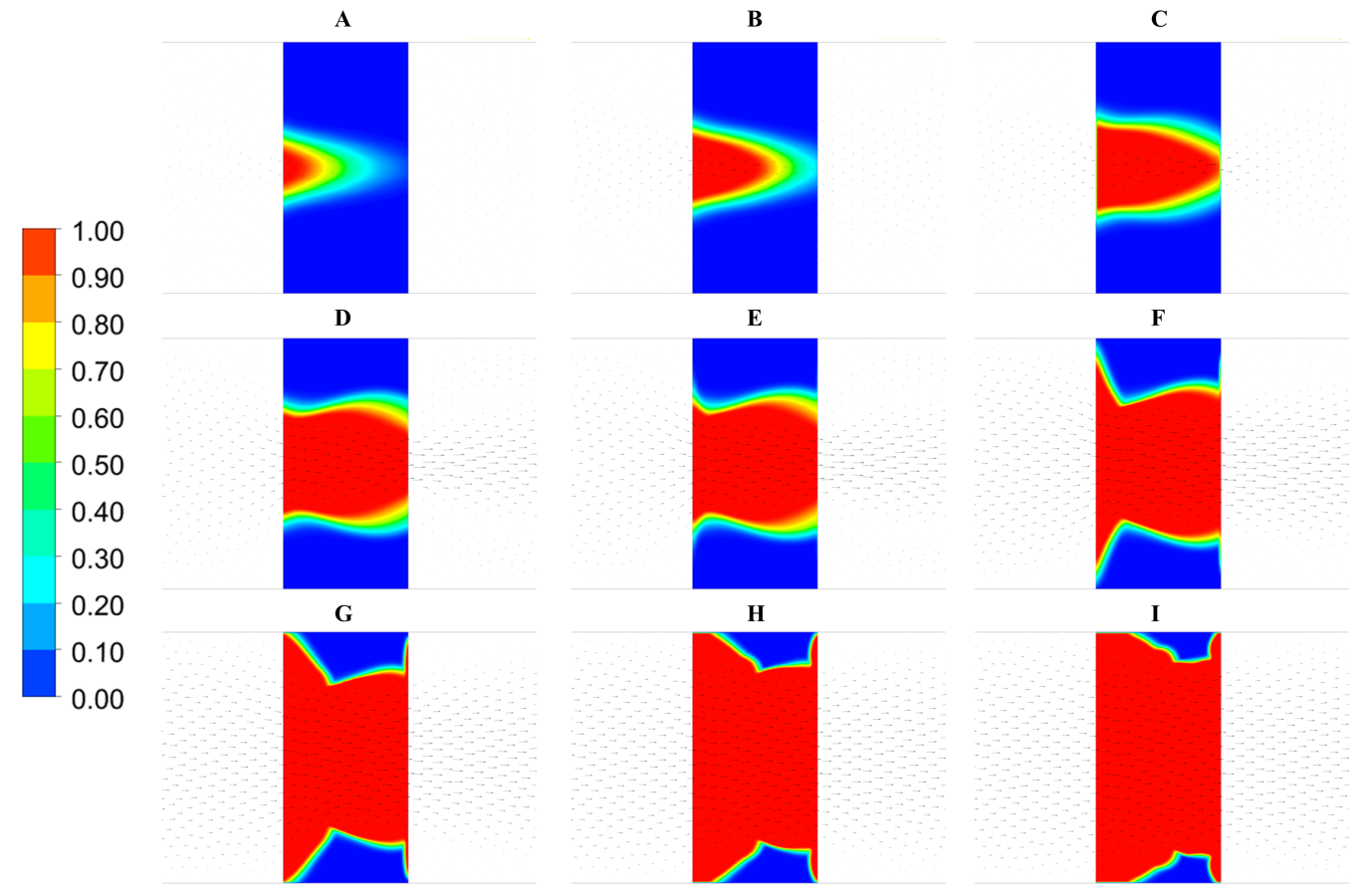


Figure A2: Change in contours of clot lysis over time for a 10 Pa pressure drop. (A) BTR = 0.50; (B) BTR = 0.75; (C) BTR = 1.00; (D) BTR = 1.25; (E) BTR = 1.50; (F) BTR = 1.75; (G) BTR = 2.00; (H) BTR = 2.25; (I) BTR = 2.50.


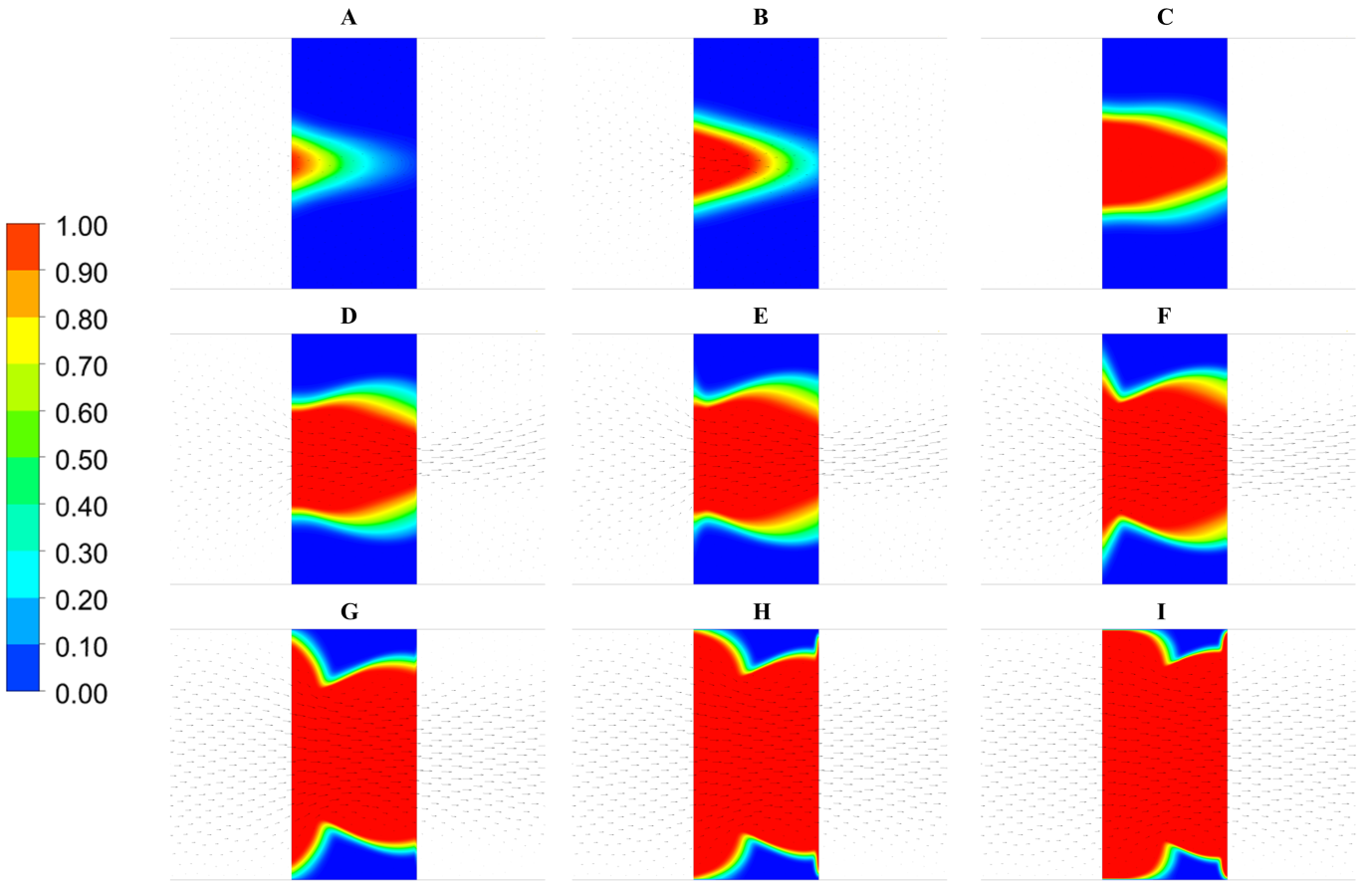


Figure A3: Change in contours of clot lysis over time for a 20 Pa pressure drop. (A) BTR = 0.50; (B) BTR = 0.75; (C) BTR = 1.00; (D) BTR = 1.20; (E) BTR = 1.40; (F) BTR = 1.60; (G) BTR = 1.80; (H) BTR = 2.00; (I) BTR = 2.20.

**EQUATION DERIVATIONS**

The following section presents derivation of some of the equations presented in Section 2. Further details can be found in the literature (6, 7, 8). In order to evaluate the change in fibrin fibre radius, the loss in fibrin due to lysis is attributed to an overall reduction in fibre radius.

|  | 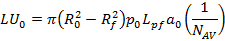 |  |
| --- | --- | --- |

In order to calculate the amount of fibrin lost, the equation evaluates the amount of protofibrils per fibre cross-sectional area, the length of these fibres and the amount of fibrin monomers on each of these protofibril strands. The units can be converted from fibrin molecules into moles by using Avogadro’s number. This expression can be rearranged to give the change of the fibrin radius as a function of the amount of fibrin lysed.

The length of the protofibrils can be evaluated by performing another fibrin mass balance but this time relating the total concentration of fibrin in the system to the amount of fibrin in the fibre.

|  | 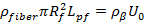 |  |
| --- | --- | --- |

This equation can be rearranged to give the following expression for the protofibril length:

|  | 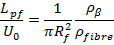 |  |
| --- | --- | --- |

Since *ρ_β_* is an input parameter in the system and *ρ_fibre_* is a constant, the initial fibre length can be evaluated. It can then be used to calculate the voidage of the control volume.

The concentration of binding sites depends on the amount of protofibrils that are accessible to the free phase species. In this study we assume that all protofibrils in the fibrin radius are accessible. This can be evaluated mathematically as a double integral over the fibre cross-section.

|  | 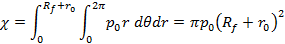 |  |
| --- | --- | --- |

The lower bound corresponds to the centre of the fibre radius (located at r = 0) while the upper bound of the integral is at the outer edge of the fibrin surface (R_f_) within one protofibril radius (r_0_).

1. Wufsus, A. R., Macera, N. E., Neeves K. B. 2013 The hydraulic permeability of blood clots as a function of fibrin and platelet density. *Biophys. J.* **104.8**, 1812-1823. [↑](#footnote-ref-2)
